# Volvulus of the ascending colon due to failure of zygosis: A case report and review of the literature

**DOI:** 10.1016/j.ijscr.2019.05.014

**Published:** 2019-05-14

**Authors:** Dennis Mazingi, Chenesa Mbanje, Godfrey I. Muguti, Taurai Zimunhu, Bothwell Mbuwayesango

**Affiliations:** College of Health Sciences, University of Zimbabwe, Parirenyatwa Hospital, Mazowe Street, P. O. Box A168, Avondale, Harare, Zimbabwe

**Keywords:** Volvulus, Peritoneal zygosis, Mesentery, Peritoneum, Mobile right colon syndrome, Intestinal fixation

## Abstract

**Introduction:**

Volvulus of the mobile ascending colon is a serious complication of a subtle embryological abnormality that frequently goes unrecognised. There has been renewed interest in the development of the mesentery because of its relevance to the contemporary management of surgical diseases. This case is presented to illustrate the dire consequences of missing this diagnosis and to review the fascinating embryology of the condition as well as its clinical implications.

**Presentation of case:**

We report on a 23-year-old male who presented with signs and symptoms of distal small bowel obstruction after a long prior history of intermittent right lower quadrant pain. At laparotomy, a 360-degree counter-clockwise volvulus of the entire right colon was noted as the result of an excessively long ascending mesocolon and unattached hepatic flexure. All gangrenous bowel was resected with primary ileocolic anastomosis performed thereafter. The patient did well post-operatively and was subsequently discharged.

**Discussion:**

Interruption of the in-utero events of fixation known as peritoneal zygosis lead to a persistence of the mesocolon in parts of the bowel that ordinarily are retroperitoneal. The events that lead to this anomaly are poorly understood and have been subject to controversy for centuries. New insights have challenged surgical dogma and informed new surgical techniques. Its true incidence is probably underestimated because of its indolent clinical prodrome, and it requires a high index of suspicion.

**Conclusion:**

Failure of peritoneal zygosis is implicated in a myriad of clinical conditions. Expeditious recognition and intervention in the prodromal period can avert potentially disastrous complications.

## Highlights

• The ascending colon has been described as a retroperitoneal organ however it may occasionally have a long mesentery.
• The process of peritoneal zygosis is incompletely understood and is subject to perturbation leading to many abnormalities.
• The excessive mobility may predispose to volvulus of this intestinal segment.
• There is often a long symptomatic prodrome before volvulus occurs during which the observant surgeon may intervene.
• The mesentery has gained new relevance with respect to its role in the contemporary management of surgical diseases.

## 1 Introduction

This case has been reported in accordance with the surgical case report guidelines (SCARE) criteria [1]. Volvulus of a mobile right colon is an infrequent complication of an uncommon embryological anomaly [2]. Patients generally have a long prior history of pain [3] that should alert the diligent physician to excessive colonic mobility. Prompt recognition may prevent disastrous consequences. Laparoscopic cecopexy is currently the preventative procedure of choice [4]. We report a case of an adult male who presented with volvulus of the entire right side of the colon and discuss the embryology as well as the clinical implications of this interesting entity.

## 2 Case report

A 23-year-old male patient was referred to our public teaching hospital with two days history of colicky periumbilical abdominal pain, feculent vomiting and obstipation for one day. He had not had prior surgery, was HIV negative and had been generally well prior to this illness. He did however, admit to recurrent right lower quadrant abdominal pain throughout his life which had never been previously investigated. He had no relevant drug, family, psychosocial or genetic history.

Upon examination, he appeared dehydrated, lethargic and in mild respiratory distress. He had a temperature of 36.9 °C, a respiratory rate of 40 breaths per minute and a saturation of 82%. He had moderate abdominal distension, visible peristaltic movements as well as peritonitis with rigidity and rebound tenderness. Digital rectal examination and assessment of other systems were unremarkable.

Blood investigations revealed leucocytosis of 13.11/mm^3^ (4–11/mm^3^), haemoglobin of 19.9 g/dl (14–17 g/dl), reflecting dehydration. He also had a high urea: 97 mMol/L (2–6.7 mMol/L) and serum sodium was 131 mMol/L (133–146 mMol/L).

An acute abdominal series of x-rays was performed which showed distended loops of small bowel with multiple air fluid levels.

A diagnosis of distal small bowel obstruction was made. The patient was immediately admitted and catheterised; resuscitation was instituted with crystalloids, intravenous antibiotics and nasogastric decompression. Oxygen was administered per face mask and he was taken to the operating room.

**Fig. 1:**
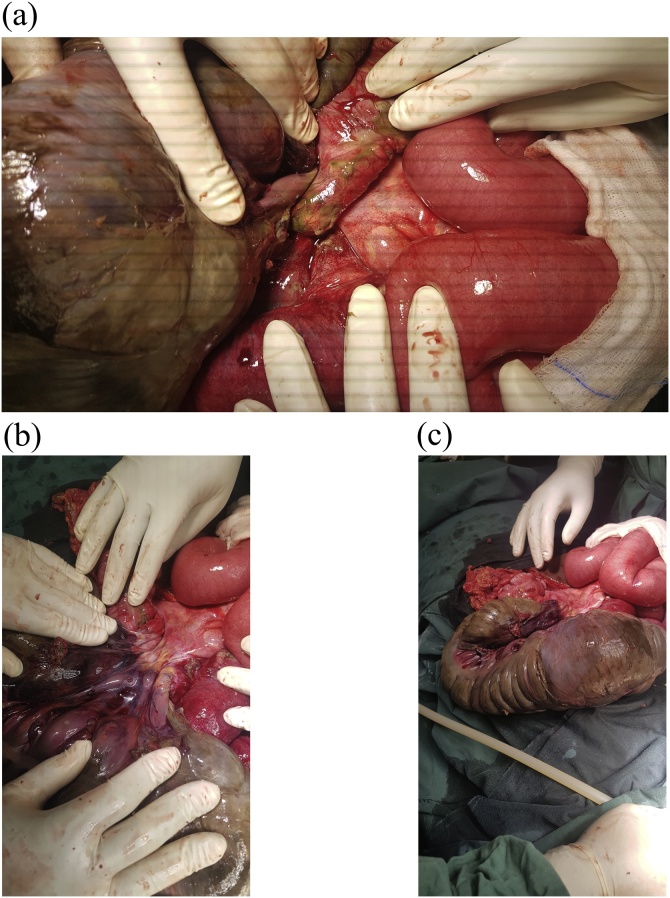
A. Showing twist of the caecum, ascending colon and hepatic flexure in a counter-clockwise direction. B shows the detorted bowel with thickened, narrow mesenteric base. C. showing the grossly dilated caecum, ascending colon and hepatic flexure in their detorted, in vivo configuration. They are sufficiently mobile that they may be lifted out of the abdomen. All of these intestinal segments were not fixed by peritoneum.

At laparotomy, haemorrhagic fluid was noted on peritoneal entry. An axial 360° counter-clockwise volvulus of the midgut from terminal ileum to mid transverse colon was encountered around a very narrow mesenteric base (Fig. 1). There was gangrene of the involved intestinal segments which appeared to be grossly distended and the hepatic flexure was noted to be unfixed and mobile (Fig. 1c). The duodenum was normally situated as was the ligament of Treitz. Right hemicolectomy was performed with primary ileocolic anastomosis and peritoneal lavage. The rest of the bowel was normal, the splenic flexure was normally fixed in the left upper quadrant and there was no distal obstruction. The operation was performed by one surgical trainee with 4 years of specialised training and one general surgeon with 3 years’ experience. Post-operatively, the patient was admitted to the intensive care unit where broad-spectrum antibiotics were continued. He did not require inotropic support and was extubated on day 3 post-operatively. He resumed oral feeds on day 4 and was eventually discharged 10 days post-operatively. At follow-up in the outpatient’s clinic 1 week later he was having an uneventful recovery with no adverse events. At further review three months later, he had no recurrence of symptoms. The patient and his family were relieved that he had done so well. Histology results confirmed gangrenous loops of bowel with no other histologically evident pathology. There are no plans for continued follow-up.

## 3 Discussion

Volvulus of the ascending colon and caecum is an infrequently encountered surgical emergency that comprises 1% of mechanical bowel obstructions [3]. It occurs as a result of an abnormality of intestinal fixation that causes persistence of the mesocolon in the normally retroperitoneal organ [3]. It was Homans in 1921 who asserted that “as a sine qua non for the development of a twist of the right colon, it must have a long mesentery” [5]. Midgut volvulus caused by classical malrotation commonly presents in childhood. However, more subtle aberrations may remain unrecognised until adulthood with potentially devastating consequences.

10-20% of people are thought to have a mobile ascending colon [6], however the true incidence is probably underestimated. Philips et al measured the mesentery of various colonic segments in cadavers and noted that two-thirds of subjects had a mobile ascending colon [7]. Additionally, Saunders et al found that ascending colon mobility was significantly more frequent in western than in oriental subjects. There is a precedent for ethnic differences in intestinal anatomy influencing the clinical picture of surgical diseases. Madiba’s seminal article describing the morphology of the sigmoid colon with regard to sigmoid volvulus found significant differences in the mesocolic ratio, sigmoid colon length, width of the sigmoid mesocolon root and frequency of the “long, narrow type” between black Africans and other ethnic groups in South Africa [8]. These differences are thought to be responsible for stark geographic, ethnic and sex differences exhibited in sigmoid volvulus. Whether this is true for the right colon is an interesting area for future research.

Abnormalities of intestinal fixation that give rise to a persistent ascending mesocolon have their origin in in-utero events during embryogenesis, what Popky called “the gyrations and migrations of the gastrointestinal tract”. These are intricately choreographed sequential events that result in elongation, rotation, migration and fixation of the foetal bowel as well as some post-natal changes until it adopts its final normal configuration. Frazer and Robbins originally used a three-stage process to describe these events [9]. Schwabe et al. contended that observed anomalies are the result of the bowel and its mesentery being “frozen” within or in-between one of the stages of development, however, the rare condition known as “reversed rotation” appears to contradict this overly simplistic view. According to Frazer and Robbins, stage 1 encompasses the process of physiologic umbilical herniation from week 5 to week 10; during stage 2 (week 10 to week 11) the midgut loop reduces back into the abdomen and stage 3 is the period when fixation occurs and lasts until shortly after birth [9]. Other authors have since described these stages differently [10]. The process of fixation of intestinal segments during stage 3 is also known as peritoneal zygosis.

Peritoneal zygosis is an abstruse process that involves adhesion of the primitive dorsal mesentery of the foetal gut with the peritoneum of the posterior abdominal wall resulting in “retroperitonealisation” of certain intestinal segments. This process of fixation occurs only in anthropoid apes and man and appears to be related to upright posture [11]. For centuries there have existed two theories to explain the events during zygosis: the sliding theory and the theory of peritoneal fusion [12]. The fusion theory has gained prominence at the expense of the sliding theory which was widely held surgical dogma originally espoused by Treves. Carl Toldt’s 1879 theory, known as the “adhesion and fixation model”, was recently given credence to by new observations that the mesentery does not actually regress but persists into adulthood, being separated from the retroperitoneum by the weakly adherent “lamina mesenteria propria” which still bears his name today (Toldt’s fascia) [13]. This fact is exploited by modern-day surgeons in achieving oncologically sound colorectal resections, the so-called “total mesocolic excision”. The degree of mobility of the colon is proportionate to the failure of attachment of the primitive mesentery to the posterior abdominal wall [14].

Balthazar postulated that excessive mobility on its own was not enough to cause volvulus of the ascending colon and that other mechanical factors were required [15]. He suggested that colonic distention and a fulcrum on which the bowel can rotate were both necessary preconditions for volvulus [15]. This is analogous to the left side of the colon with regard to sigmoid volvulus. Madiba in 2011 contended that a narrow mesenteric base as well as a long, narrow, redundant sigmoid colon predisposed to sigmoid volvulus [8]. In our case, the mesenteric base was noted to be very narrow and the involved intestinal segment was markedly distended.

Excessive mobility of the right colon has been linked with several clinical problems. Volvulus, as exhibited in our case, represents the extreme in terms of severity. However, patients may also present with a spectrum of non-specific chronic right lower abdominal pain that has been called the “mobile right colon syndrome” [14] thought to be due to repeated twisting and untwisting of the bowel or traction on the superior mesenteric artery [15]. It may also present as right-sided thrust dyspareunia. Kanai and colleagues implicated excessive colonic mobility in colonic varices thought to be the result of local venous hypertension caused by repeated extrinsic vascular impingement [16]. Another postulated consequence of a mobile ascending colon is intussusception [17]. Waugh was the first to describe the phenomenon of intussusception in combination with malrotation [18] and Brereton in his classic series found that every patient with intussusception had at least some form of disorder of intestinal rotation on the spectrum from overt classical malrotation to subtle mobility of the ascending colon [19]. He suggested that mobility was a risk factor or even a necessary precondition for intussusception [19]. Mobility may also facilitate significant distal migration of the intussusceptum even as far as rectal protrusion [20]. Lastly, excessive mobility of the ascending colon may predispose them to herniation into the lesser sac through the foramen of Winslow, or into femoral or inguino-scrotal hernial sacs [21]. Despite the multitude of conditions linked to right coloptosis, there are limits to the complicity of the ascending colon. In his treatise describing the morbid consequences of the mobile right colon, Waugh appeared to attribute nearly every abdominal malady to the mobility of the ascending colon including gastric ulcers, gallstone disease and various renal ailments [18]. Alternative explanations have since been elucidated for these conditions.

## 4 Conclusion

Persistent ascending mesocolon represents a failure of zygosis during embryological development that may present with volvulus of the midgut in adulthood. General surgeons must keep this condition in their differential when a patient has recurrent abdominal pain. Prompt diagnosis and management may allow for preventative manoeuvres to salvage bowel.

## Conflicts of interest

Nil.

## Funding

Nil.

## Ethical approval

Ethical approval for this publication has been exempted by our institution.

## Consent

Written informed consent was obtained from the patient for publication of this case report and accompanying images. A copy of the written consent is available for review by The Editor-in-Chief of this journal on request.

## Author’s contribution

Dennis Mazingi - case report design, subject research, consent and writing.

Chenesa Mbanje - case report design, writing, research and editing.

Godfrey Muguti - case report design and editing.

Taurai Zimunhu - case report design, writing, research and editing.

Bothwell Mbuwayesango - case report design.

## Registration of research studies

N/A.

## Provenance and peer review

Not commissioned, externally peer-reviewed.
